# Efficacy of Seabuckthorn (*Hippophae rhamnoides*) Oil *vis-a-vis* Other Standard Drugs for Management of Gastric Ulceration and Erosions in Dogs

**DOI:** 10.1155/2013/176848

**Published:** 2013-06-18

**Authors:** Richa Dogra, S. P. Tyagi, Amit Kumar

**Affiliations:** Department of Surgery and Radiology, Dr. G. C. Negi College of Veterinary and Animal Sciences, CSKHPKV, Palampur 176062, India

## Abstract

The study was conducted on 20 adult healthy medium-sized mongrel dogs. Injection of dexamethasone @ 1 mg/kg, IV, b.i.d., was administered to create gastric ulcerations and erosions. Thereafter all the animals were randomly divided into 5 equal treatment groups. Animals of groups I, II, III, IV, and V were treated with oral administration of lansoprazole @ 1.5 mg/kg, sucralfate @ 1 g/animal, misoprostol @ 10 *µ*g/kg, famotidine @ 1 mg/kg, and Seabuckthorn seed oil @ 5 mL/animal, twice a day, respectively. Gastroendoscopically, complete healing of GUE lesions was earliest in Seabuckthorn- (SBT-) oil-treated group (7.5 ± 0.87) followed by famotidine (8.25 ± 1.44), lansoprazole (9.00 ± 1.23), misoprostol (10.50 ± 1.50), and sucralfate (13.50 ± 0.87), respectively. A marked improvement in appetite was observed in all animals. Melena was continued till day 3 in SBT group, day 6 in lansoprazole- and famotidine-treated animals, and day 9 in sucralfate and misoprostol group animals. Fecal occult blood test was positive in all animals till there was endoscopic evidence of gastric bleeding. Hematological parameters improved markedly towards the end of the study. Serum biochemical parameters remained within normal physiological limits throughout the study. It is concluded that Seabuckthorn oil was the best therapeutic agent for dexamethasone-induced GUE in dogs followed by famotidine, lansoprazole, misoprostol, and sucralfate.

## 1. Introduction

Gastric ulcerations and erosions (GUEs) are a well-known entity in veterinary medicine. The mucosal defect penetrating through the gastric muscularis mucosa is termed as “gastric ulcer,” whereas the superficial ulcer that does not extend far into the mucosa is termed as “gastric erosion.” But in routine clinical practice, it is difficult to differentiate between both conditions by all known diagnostic methods except histopathology. So this complex of gastric ulcerations and erosions is combined termed as GUE. In small animals, it develops mainly due to long-term administration of steroidal and nonsteroidal anti-inflammatory drugs (NSAIDs). Corticosteroids are ulcerogenic in dogs even at therapeutic doses [1]. Ulcerogenic activity of these drugs is attributed to their inhibitory effect on synthesis of prostaglandins, altering the biochemical structure of gastric mucous which increases acid output. This exposes the gastric wall to its own acids leading to GUE. Other potential causes of gastric ulceration in animals include neoplasia like lymphosarcoma, adenocarinomas, gastrinoma (Zollinger-Ellison syndrome), and mastocytosis, systemic diseases like hepatic and renal disease, hypovolemic shock, hypoadrenocorticism, sepsis, spinal injury, and pancreatitis, infectious agents like *Helicobacter* species, pyloric outlet obstruction, inflammatory bowel disease, and chronic gastritis.

 Medical management of GUE aims to neutralize or inhibit the gastric acid secretion and supports the integrity of gastric mucosa. Therefore, standard medical management of GUE comprises antacids, proton pump inhibitors, H_2_-receptor antagonists, and cytoprotectant drugs. In small animal practice, all these drugs are used but their doses and frequencies are mostly extrapolated from human medicines. There is very limited research to standardize the doses and frequencies of these drugs in veterinary medicine. Consequently, these medicines have questionable efficacy along with unknown side effects in small animals. A lot of research is going on globally to find out alternative medicines including herbal preparations having better efficacy and lesser side effects for management of GUE in dogs.

Many herbal preparations like Licorice root [2], *Aloe vera* [3], *Picrorhiza kurroa* [4], *Ginkgo biloba* [5], Saffron [6], *Jatropha curcas *[7], Cinnamon and Chamomile [8],  *Nigella sativa* [9], and Seabuckthorn [10] are considered to possess antigastroulcerative properties. 

Of  late, Seabuckthorn (*Hippophae rhamnoides*) has drawn the attention of scientists because of its multifarious medicinal properties including the antigastroulcerative one. The different preparations of Seabuckthorn such as decoction, powder, pill, medicinal extract, ash, and tincture have been used for the treatment of various disease conditions in ancient times [11]. Clinical trials and scientific studies conducted during the last few decades in several countries like China, Russia, and India confirmed its medicinal and nutritional value. It is known to possess anti-inflammatory [12], hepatoprotective [13], anticancerous, antilipemic, antiarrhythmic, cutaneous wound healing [15], burn wound healing [16], and antigastroulcerative activity [17]. The research on the biochemical composition of Seabuckthorn has revealed that it contains many kinds of vitamins, trace elements, amino acids, and a number of bioactive substances which are responsible for its versatile pharmacological activities. Of all types of Seabuckthorn preparations, its oil is primarily known to improve the conditions of mucous membranes, including ulcers and erosions, and is thus reported to have antigastroulcerative properties. Xing et al. [18] reported that Seabuckthorn oil reduces ulcer formation in experimental gastric ulcer model in rats. Süleyman et al. [10] reported that the gastric ulcer healing properties of Seabuckthorn oil are better than omeprazole in rats. Tyagi [17] reported that Seabuckthorn oil can reduce the severity of GUE lesions being induced by long-term use of dexamethasone in dogs. However, a systematic study to compare various standard gastric ulcer drugs with Seabuckthorn oil in animals is still lacking. Therefore, this study design was made to comparatively evaluate the therapeutic potential of Seabuckthorn (*Hippophae rhamnoides*) oil with routinely used standard medicines in gastric ulceration and erosion (GUE) in dogs.

## 2. Materials and Methods

For the study a number of random sourced adult mongrel dogs of either sex were screened for their health status by subjecting them to routine clinical and hematological examinations. Out of these, 20 average sized dogs weighing 15–25 kg and having hematological parameters within the normal range were eventually utilized in the study. These dogs were kept in kennels of for acclimatization for at least 10 days prior to the start of trials. The animals were kept in individual pans in well-ventilated and thermocontrolled kennel. The kennel is well equipped with 24-hour water and power supply. They were fed balanced commercially available best quality dog feed and had unrestricted access to clean drinking water. All the animals were treated for internal and external parasites and vaccinated against rabies on the first day itself. Dogs were kept under standard and uniform managerial conditions. *Requisite prior permission for experimentation for the study was duly obtained from institutional animal ethics committee.*

For the experimental creation of nonfatal GUE Inj. dexamethasone was administered in all the dogs @ 1 mg/kg, IV, b.i.d. until there was endoscopic evidence of GUE reaching to ulcer index 7 on two consecutive endoscopic observations or 8 in a single observation occasion as per Tyagi [19]. Afterwards, the animals were randomly divided into 5 equal treatment groups (*n* = 4). Animals of various groups were treated with oral administration of lansoprazole @ 1.5 mg/kg (group I), sucralfate @ 1 g/animal (group II), misoprostol @ 10 *μ*g/kg (group III), famotidine @ 1 mg/kg (group IV), and Seabuckthorn seed oil @ 5 ml/animal (group V), twice a day, respectively, till complete healing of GUE.

The development of gastric lesions and their healing were evaluated by clinical parameters like rectal temperature (°F), heart rate (/min), respiration rate (/min), colour of mucous membrane (cmm), body weight (kg), variations in appetite, vomiting, colic, melena, diarrhea, constipation, any change in hair coat and skin or any other behavioural change in dogs, hematological parameters like hemoglobin (Hb), packed cell volume (PCV), total erythrocyte count (TEC), total leukocyte count (TLC), and differential leukocyte count (DLC), biochemical parameters such as aspartate transaminase (AST), alanine transaminase (ALT), total protein (TP), blood urea nitrogen (BUN), and creatinine (CRTN), Fecal occult blood test (FOBT), and gastroendoscopic examinations. During gastroendoscopic examinations all the areas of stomach, namely, fundus, gastric body, and pylorus, were examined for GUE lesions. GUE index was determined on the basis of the number of gastric lesions and severity scoring system as per Tyagi [17].

*Fecal occult blood test (FOBT)* was done to detect the presence of digested blood in faeces which might be otherwise not visible to naked eyes as per the method of Oser [20]. The biochemical analysis was done at weekly intervals, whereas the other examinations were performed at 0, 4, 7, 10, 13, 16, 19, 22, and 25 days. Statistical analysis of data was carried out using analysis of variance (ANOVA) method at 5% and 1% levels of significance using Students-Newman-Keuls test of InStat software (GraphPad).

## 3. Results and Discussion

### 3.1. Gastroendoscopic Findings

**Figure 1:**
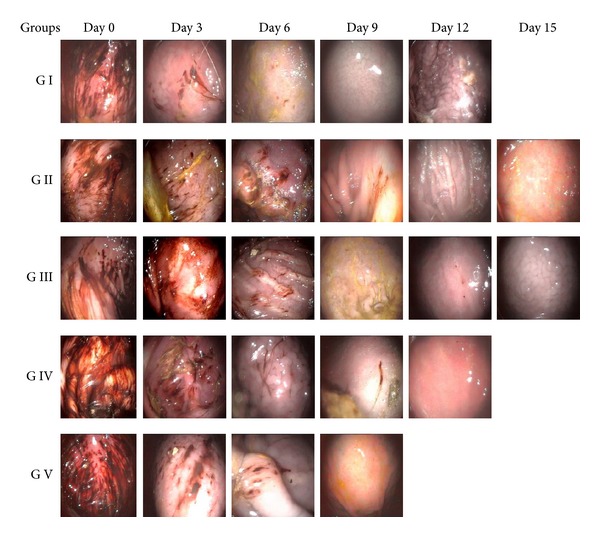
Endoscopic view of gastric mucosal surface of dogs in different groups at various observation intervals.

 On day 0, the severity of gastric lesions was maximum, and GUE indices were 8.00 ± 0.00, 7.75 ± 0.25, 7.25 ± 0.25, 7.75 ± 0.25, and 7.50 ± 0.29 in groups I, II, III, IV, and V, respectively (Tables 1 and 2, Figure 1). In general, the lesions were largely similar in all animals and consisted of multiple linear as well as focal mucosal defects of variable shapes and depths. The lesions were generally larger and widely distributed all over the gastrum, that is, fundus, body, and pylorus. Large adherent multiple blood clots as well as fresh blood were observed inside stomachs of all the dogs. The mucosa was severely hyperemic and the mucus layer was appreciably very thin (Figure 1). On day 3, in group I, the GUE index was 5.00 ± 0.00 and the gastric lesions consisted of small multifocal lesions with adherent as well as free floating blood clots primarily over gastric body and pylorus. In group II, that is, sucralfate-treated animals, the mean GUE index was 5.25 ± 0.25. Mucosa was thin in all animals, although submucosal engorged blood vessels were evident in only one animal. In group III, that is, misoprostol-treated animals, trivial improvement in the gastric mucosa was observed as compared to other groups. GUE index was 6.75 ± 0.25 which was quite near to day zero. The greatest reduction in mean GUE index was observed in group IV, that is, famotidine-treated animals, at this interval. The GUE index was 4.75 ± 0.63, but a marked individual variation in GUE index of animals of this group ranging from 3.00 to 6.00 was observed. In group V, that is, Seabuckthorn-oil-treated dogs, the mean GUE index was 5.00 ± 0.82 with an even greater individual variation ranging from 3.00 to 7.00. The decrease was statistically significant within each group but insignificant in between groups. The mucus layer was best as evidenced by its thick shiny nature in group IV followed by group V. On day 6, GUE index fell down even more in all the groups compared to the 3rd day levels. The GUE index was least in group I and greatest in group II; it was in between in the rest of the groups. Though reduction in GUE index was statistically significant within all groups, it was insignificant in between groups. Wide individual variations were observed within groups as much as complete healing was observed in 1 animal at this stage in groups I and III and in 2 animals of groups IV and V each. In general the mucus layer was much thicker and shiny in groups IV and V. On day 9, further reduction in GUE index was observed in all groups. In group V, the healing of GUE lesions was complete and the index came down to 0. In groups I and IV also the healing was very good and the GUE indices were 0.66 ± 0.66 and 1.00 ± 1.00, respectively. In group I, 2 more animals showed complete healing of gastric lesions. The gastric mucosal surfaces were closer to normal in appearance in all dogs, but still it was not as shiny and glistening as normal mucosal layer. In group II, small, superficial, focal multiple mucosal defects were still visible. Submucosal haemorrhages were also observed in 2 animals, and the mucosal layer was not well formed and was rather thin. In group III, the mean GUE index was highest among the groups at 3.33 ± 1.33, and multiple punctuate lesions with haemorrhagic streaks were still visible. In group IV, one animal showed complete healing of GUE, but small, superficial linear lesions were still observed sporadically in one remaining dog. Overall the mucosal layer was qualitatively much better in groups IV and V.

On day 12, complete healing was observed in groups I and IV and the GUE index came to 0, whereas in group II 3 out of 4 animals showed complete healing of gastric lesions and 1 animal showed mucosal defects as few tiny, multifocal superficial lesions without active hemorrhagic base but with free floating tiny blood clots. In group III, 2 animals revealed complete recovery of gastric mucosa, but few gastric erosive defects without blood clots were still visible in one animal. The mean GUE index continued to be higher in this group, that is, 1.0 ± 1.0 with a range of 3.00 to 0.00. On day 15, though all the remaining animals in group II and group III also showed complete healing of GUE, normal mucous layer was still not appreciable in group II as compared to the rest of the groups.

**Figure 2:**
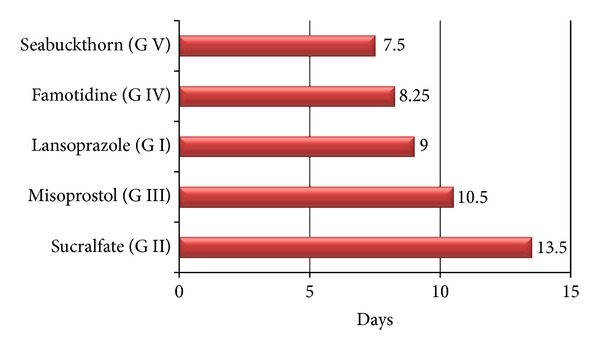
Average number of days to achieve 0 GUE index in different groups.

The complete healing of GUE lesions occurred in Seabuckthorn-oil-treated group in 7.50 days as compared to 8.25 days in group IV, 9.00 days in group I, 10.50 days in group III, and 13.50 days in group II (Figure 2). Antigastro-ulcerative property of SBT observed is in agreement with previous studies on rats, dogs [10, 17, 18, 21–26], and humans [27, 28]. It has been found effective against various kinds of gastric ulcers induced by physically necrotizing agents, NSAIDs or stress. Jiang et al. [21] identified an antiulcer component of SBT oil, that is, *β*-sitosterol-*β*-D-glucoside which significantly decreased the size of the ulcer area in their studies in certain kinds of ulcers. There are pieces of evidence, showing that the effects of  Seabuckthorn oils might be related to their antioxidative activity [10, 29–31].

On the basis of some indirect evidence, Kallio and Baoru [32] suggested that the positive effects of  Seabuckthorn oils, especially seed oil, on peptic ulcer may be related to modification of the prostaglandin synthesis of gastric or duodenal mucosa. This seems plausible as restoration of gastric mucosal layer was quick in Seabuckthorn-oil-treated group in the present study. Healing of GUE in Seabuckthorn oil group was followed by the famotidine-treated group and in both the mucosal layer was qualitatively better than other groups. The healing of GUE lesions was faster in famotidine-treated animals compared to lansoprazole-treated animals in the present study, but many authors have reported that proton pump inhibitors are more effective in gastric ulcer healing than H_2_-receptor antagonists [33–37]. However, Hotz et al. [38] reported that pain relief and the decrease of concomitant antacid consumption were comparable in both famotidine- and lansoprazole-treated animal groups. Sucralfate-treated animals showed full recovery from GUE lesions lastly among all the groups. Since without treatment also the spontaneous healing of GUE lesions usually takes place within 12–16 days in dogs [19], the therapeutic potential of sucralfate is debatable in present model of GUE because healing took on an average 13.50 days in this group in the present study. Borne and MacAllister [39] also reported that sucralfate @ 22 mg/kg PO for 14 days, every 8 hours, did not promote greater healing than did the corn syrup in foals. 

### 3.2. Clinical Parameters

A gradual decrease in rectal temperature, respiration rate, and heart rate was observed in all the groups. The rectal temperature, respiration rate, and heart rate did not vary much with the base values and remained within normal physiological limits throughout the period of study in all the groups. No statistical difference was observed between various groups at any observation intervals (Table 3).

The healing process of gastric mucosa under various treatment regimens resulted into inhibition or neutralization of gastric acid secretions ultimately supporting the integrity of gastric mucosa. As a result blood losses from stomach decreased markedly which resulted in restoration of normal clinical parameters.

A nonsignificant gain of body weight was observed in the dogs of groups (Table 4). The maximum gain in body weight was 3.64%, 4.64%, 4.22% 0.87%, and 6.44% in groups I, II, III, IV, and V, respectively. Therefore, gain in weight was highest in group V followed by group II, group III, group I, and then group IV. Highest weight gain in Seabuckthorn oil group is justified due to faster healing as evidenced endoscopically over different observation intervals and rapid restoration of digestive processes.

A marked improvement in appetite was observed in all animals during treatment. Most of the animals started showing improvement 3 days after the start of treatment, but two animals continued with decreased appetite till the 9th day in group II. Towards the end of the study all the animals had regained their normal appetite. During treatment no vomiting and diarrhoea were observed in any of the animals, but melena was observed till day 3 in group V, day 6 in group I and group IV, whereas it continued to be seen till 9th day in group II and group III. The severity of melena gradually decreased towards the end of study in all the groups. Skin lesions were observed in two animals in group II, two animals in group III, one animal in group IV, and one in group V on day 0, but further development of skin lesions was not observed in any animal in any of the groups.

### 3.3. Fecal Occult Blood Test (FOBT)

**Figure 3:**
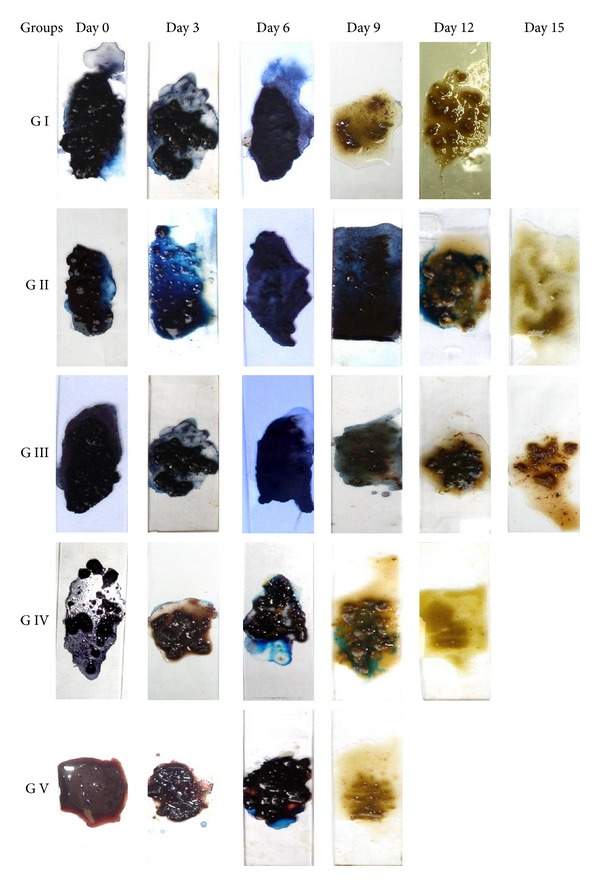
Representative fecal occult blood test reactions of dogs in different groups at various observation intervals.

The fecal occult blood test was strongly positive in all the groups at day 0. Thereafter, the strength of FOBT reactions gradually decreased but varied within and in between various groups (Figure 3). On all the instances a direct correlation was observed between detection of blood clots or gastric lesions endoscopically and a corresponding FOBT reaction. No false positive or false negative reaction was observed at any intervals. This indicated that fecal occult blood test is proficient in diagnosing smaller quantities of blood in faeces in cases of subclinical GUE in dogs. Detection of gastric bleeding even in minute quantities endoscopically corresponded to FOBT reaction every time. This indicated that fecal occult blood test is proficient in diagnosing smaller quantities of blood in faeces in cases of subclinical GUE in dogs. Gilson et al. [40] reported that fecal occult blood tests could detect quantities of blood that were smaller than those required to cause melena. Rohrer et al. [41] detected occult blood in high percentage of dogs (9/10) in which gastric haemorrhages was evident after administration of methylprednisolone sodium succinate.

### 3.4. Hematological Parameters

 In general, a gradual rise in Hb, PCV, and TEC levels was observed from 0 day till the end of study in all the groups except group II (Table 5). However, the rises were statistically insignificant within as well as in between groups. In group II, Hb, PCV, and TEC continued to drop till the 6th day but started rising thereafter. Moreover, in group IV, the PCV increased on day 3 but decreased slightly on day 6. This was mainly because of the variation in one animal of this group that did not show any improvement in PCV; rather a continuous decline was observed. PCV improved earliest in group V followed by groups I and IV and lastly II and III. In group II, that is, sucralfate group, recuperation in TEC started on day 9 rather than day 3 as observed in other groups.

TLC and granulocytes decreased in all the groups over different observation intervals, but the decrease in TLC was significant on the 6th, 9th, and 12th days in group II, 3rd, 6th, 9th, and 12th days in group III, and 3rd and 6th days in groups IV and V. Similarly, significant decrease in granulocytes was observed on the 6th, 9th, and 12th days in group III. Towards the end of study lymphocytes and monocytes increased in all groups, but the increase in lymphocytes was significant on days 6 and 9 in group V only. No statistically significant variations were observed within different groups in TLC and DLC (Tables 6 and 7).

Lansoprazole, sucralfate, misoprostol, famotidine, and Seabuckthorn oil are safe to administer in dogs as these drugs did not resulted in any adverse effect on haematological as well as biochemical parameters in any of the groups. Jensen et al. [42] reported that lansoprazole @ 60 mg/day for 31 days did not produce any significant changes in haematological parameters in human patients with Zollinger-Ellison syndrome. Similarly, Hentschel et al. [43] reported that hematological parameters were not affected by treatment with sucralfate @ 1 g, PO, thrice a day, in endoscopically diagnosed duodenal ulcer patients. Furthermore, Robinson and Sly [44] reported that following administration of misoprostol @ 100 *μ*g, PO, q.i.d., for the treatment of cystic fibrosis, one patient had a significant elevation in the eosinophil count, but there were no significant changes in any other hematological parameters. Similarly, Tyagi [17] reported a gradual increase in Hb, PCV, and TEC following administration of Seabuckthorn seed oil, at the same dose rate used in the present study, in dexamethasone-induced GUE in dogs.

### 3.5. Biochemical Parameters

 Biochemical parameters were recorded from the day of the start of dexamethasone to induce nonfatal GUE unlike other parameters which were recorded from the start of treatment. AST level remained elevated than base values and decreased subsequently towards the end. The variations were, however, insignificant in all groups at various observation intervals. The AST levels first increased from base value till the 7th day and decreased thereafter in groups I and II and from the 14th day onwards in groups I, IV, and V. There were great individual variations in ALT levels of dogs within groups at all observation intervals. In general, ALT levels remained elevated than their base values at all subsequent observation intervals but with no statistical significance. The patterns of variation in ALT values were again dissimilar in different groups. In group I, ALT values initially increased at the 7th day and gradually decreased till the 21st day. In group II, ALT values increased till day 7 and then decreased on day 14 but again increased thereafter. In group III, ALT values continued to increase till the last observation interval, that is, 21st day, whereas in group IV and group V, ALT levels increased gradually till the 14th day and decreased thereafter on 21st day. The value of ALT was however significant on day 21 between different groups (Table 8). 

BUN and CRTN levels of dogs in all the groups did not vary much with the base values of day 0 and remained within normal physiological limits throughout the period of study. The total protein gradually decreased in all the groups towards the end of study except in group I and group V where it remained almost near to base value towards the end. Maximum drop in total protein was revealed by group IV. In group II mild hypoproteinemia was observed. No statistical difference was observed between various groups at any observation period, but intragroup significant decrease was observed in groups II, III, and IV at various observation intervals.

## 4. Conclusion

Therefore the overall therapeutic efficacy of Seabuckthorn seed oil in dexamethasone-induced gastric ulcerations and erosions in dogs is better than famotidine, lansoprazole, misoprostol, and sucralfate. 

## Figures and Tables

**Table 1:** GUE score card.

Score	Description
*Gastric lesion number score *	
0	No lesions
1	1-2 localized lesions
2	3–5 localized lesions
3	6–10 lesions
4	>10 lesions/very large/diffuse lesion

*Gastric lesion severity score *	
0	No blood clots
1	Free floating or adherent smaller blood clots with no detectable haemorrhage base
2	(i) Adherent smaller blood clots with active haemorrhage base (ii) Apparently superficial smaller focal mucosal erosion (<3 mm) with or without active hemorrhage (iii) Apparently superficial linear mucosal erosion without active haemorrhage (iv) Submucosal hemorrhages or erythematous mucosa (v) Adherent larger blood clots without active haemorrhage base
3	(i) Apparently superficial larger focal mucosal erosion (>3 mm) with or without active haemorrhage (ii) Linear erosions with active bleeding (iii) Adherent larger blood clots with active haemorrhage base (iv) Apparently deeper mucosal lesions without haemorrhage
4	Apparently deeper mucosal lesion/ulcer with adherent large blood clots or with active haemorrhage

GUE index = lesion number score + lesion severity score	

In case of mixed lesions as per the above description, always a high score was assigned.

**Table 2:** GUE indices of dogs of different groups at various observation intervals (mean ± S.E.).

Groups	Days					
	0	3	6	9	12	15
Group I	8.00 ± 0.00	5.00** ± 0.00	2.00** ± 0.71	0.66** ± 0.66 (*n* = 3)	0.00 ± 0.00 (*n* = 1)	—
Group II	7.25 ± 0.25	5.25** ± 0.25	3.50** ± 0.29	2.75** ± 0.48	0.50** ± 0.50	0.00 ± 0.00 (*n* = 1)
Group III	7.75 ± 0.25	6.75 ± 0.25	3.25* ± 1.25	3.33* ± 1.33 (*n* = 3)	1.00** ± 0.00 (*n* = 3)	0.00 ± 0.00 (*n* = 1)
Group IV	7.50 ± 0.29	4.75 ± 0.63	2.25** ± 1.32	1.00 ± 1.0 (*n* = 2)	0.00 ± 0.00 (*n* = 1)	—
Group V	7.75 ± 0.25	5.00 ± 0.82	2.50** ± 1.44	0.00 ± 0.00 (*n* = 2)	—	—

*P < 0.05, **P < 0.01.

**Table 3:** Rectal temperature, heart rate, and respiration rate in different groups at various intervals (mean ± S.E.).

Groups	Days					
	0	3	6	9	12	15
Rectal temperature (°F)						
Group I	101.58 ± 0.10 (*n* = 4)	101.2 ± 0.39 (*n* = 4)	101.3 ± 0.06 (*n* = 4)	101.47 ± 0.06 (*n* = 3)	101.2 ± 0.00 (*n* = 1)	—
Group II	101.3 ± 0.21 (*n* = 4)	101.33 ± 0.23 (*n* = 4)	101.25 ± 0.13 (*n* = 4)	101.07 ± 0.19 (*n* = 4)	100.85 ± 0.19 (*n* = 4)	100.8 ± 0.00 (*n* = 1)
Group III	101.3 ± 0.37 (*n* = 4)	101.25 ± 0.41 (*n* = 4)	101.03 ± 0.26 (*n* = 4)	101.2 ± 0.23 (*n* = 3)	100.73 ± 0.29 (*n* = 3)	100.7 ± 0.10 (*n* = 1)
Group IV	101.05 ± 0.15 (*n* = 4)	101.05 ± 0.15 (*n* = 4)	101 ± 0.24 (*n* = 4)	101.4 ± 0.20 (*n* = 2)	101.2 ± 0.00 (*n* = 1)	—
Group V	101.02 ± 0.25 (*n* = 4)	101.23 ± 0.06 (*n* = 4)	101.15 ± 0.09 (*n* = 4)	100.9 ± 0.10 (*n* = 2)	—	—

Heart rate (per min)						
Group I	85.5 ± 3.30 (*n* = 4)	83 ± 3.12 (*n* = 4)	82 ± 2.71 (*n* = 4)	85.33 ± 3.71 (*n* = 3)	83 ± 0.00 (*n* = 1)	—
Group II	89.5 ± 0.96 (*n* = 4)	88.5 ± 2.22 (*n* = 4)	87.5 ± 2.22 (*n* = 4)	85 ± 2.08 (*n* = 4)	83 ± 1.29 (*n* = 4)	80 ± 0.00 (*n* = 1)
Group III	90.5 ± 5.91 (*n* = 4)	88.25 ± 5.66 (*n* = 4)	88.25 ± 4.80 (*n* = 4)	80 ± 2.00 (*n* = 3)	84.33 ± 1.86 (*n* = 3)	80.5 ± 5.5 (*n* = 1)
Group IV	90.5 ± 3.3 (*n* = 4)	88.5 ± 3.78 (*n* = 4)	88.5 ± 0.50 (*n* = 4)	84 ± 2.00 (*n* = 2)	84 ± 0.00 (*n* = 1)	—
Group V	91 ± 2.65 (*n* = 4)	87 ± 0.29 (*n* = 4)	87.5 ± 3.4 (*n* = 4)	87 ± 1.00 (*n* = 2)	—	—

Respiration rate (per min)						
Group I	42 ± 3.56 (*n* = 4)	42.5 ± 3.76 (*n* = 4)	41.25 ± 5.02 (*n* = 4)	40.67 ± 4.06 (*n* = 3)	38 ± 0.00 (*n* = 1)	—
Group II	35.5 ± 4.19 (*n* = 4)	35 ± 4.36 (*n* = 4)	33 ± 4.36 (*n* = 4)	31 ± 3.00 (*n* = 4)	29.5 ± 2.87 (*n* = 4)	34 ± 0.00 (*n* = 1)
Group III	37.25 ± 1.80 (*n* = 4)	37.25 ± 3.64 (*n* = 4)	33.5 ± 0.96 (*n* = 4)	31.33 ± 1.76 (*n* = 3)	28.66 ± 1.33 (*n* = 3)	28 ± 2.00 (*n* = 1)
Group IV	40 ± 3.37 (*n* = 4)	38.75 ± 5.5 (*n* = 4)	35.5 ± 4.5 (*n* = 4)	40 ± 2.00 (*n* = 2)	40 ± 0.00 (*n* = 1)	—
Group V	30.5 ± 2.22 (*n* = 4)	31.5 ± 1.26 (*n* = 4)	27 ± 1.73 (*n* = 4)	30 ± 4.0 (*n* = 2)	—	—

**Table 4:** Body weight in dogs in different groups at various intervals (mean ± S.E.).

Groups	Days					
	0	3	6	9	12	15
Body weight (kg)						
Group I	19.23 ± 2.75 (*n* = 4)	19.36 ± 2.77 (*n* = 4)	19.85 ± 2.77 (*n* = 4)	19.93 ± 3.87 (*n* = 3)	26 ± 0.00 (*n* = 1)	—
Group II	16.38 ± 1.77 (*n* = 4)	16.54 ± 1.98 (*n* = 4)	16.66 ± 2.08 (*n* = 4)	16.94 ± 1.94 (*n* = 4)	17.14 ± 1.94 (*n* = 4)	16.02 ± 0.00 (*n* = 1)
Group III	17.55 ± 1.62 (*n* = 4)	17.68 ± 1.60 (*n* = 4)	17.69 ± 1.49 (*n* = 4)	18.20 ± 1.95 (*n* = 3)	18.29 ± 1.90 (*n* = 3)	20.06 ± 0.00 (*n* = 1)
Group IV	17.22 ± 0.95 (*n* = 4)	17.22 ± 1.08 (*n* = 4)	17.32 ± 1.06 (*n* = 4)	17.37 ± 1.88 (*n* = 2)	16 ± 0.00 (*n* = 1)	—
Group V	20.97 ± 1.32 (*n* = 4)	20.96 ± 1.37 (*n* = 4)	22.32 ± 1.26 (*n* = 4)	24.55 ± 0.53 (*n* = 2)	—	—

**Table 5:** Hemoglobin, packed cell volume, and total erythrocyte counts of different groups at various observation intervals (mean ± S.E.).

Groups	Days					
	0	3	6	9	12	15
Hb (g/dL)						
Group I	11.05 ± 0.82 (*n* = 4)	12.17 ± 0.67 (*n* = 4)	12.77 ± 0.69 (*n* = 4)	12.93 ± 0.96 (*n* = 3)	14.3 ± 0.00 (*n* = 1)	—
Group II	10.75 ± 1.27 (*n* = 4)	10.05 ± 1.38 (*n* = 4)	9.75 ± 1.01 (*n* = 4)	10.6 ± 0.83 (*n* = 4)	11.9 ± 0.36 (*n* = 4)	13.4 ± 0.00 (*n* = 1)
Group III	8.73 ± 1.41 (*n* = 4)	9.97 ± 1.24 (*n* = 4)	10.05 ± 1.29 (*n* = 4)	9.3 ± 0.40 (*n* = 3)	9.64 ± 0.38 (*n* = 3)	9.8 ± 0.00 (*n* = 1)
Group IV	11.63 ± 0.42 (*n* = 4)	11.83 ± 0.56 (*n* = 4)	11.95 ± 1.89 (*n* = 4)	9.15 ± 3.65 (*n* = 2)	6.5 ± 0.00 (*n* = 1)	—
Group V	12.80 ± 0.53 (*n* = 4)	13.10 ± 0.64 (*n* = 4)	13.83 ± 0.65 (*n* = 4)	14.45 ± 0.25 (*n* = 2)	—	—

PCV (%)						
Group I	29.15 ± 2.01 (*n* = 4)	31.73 ± 1.42 (*n* = 4)	34.25 ± 1.56 (*n* = 4)	35.13 ± 1.33 (*n* = 3)	35.20 ± 0.00 (*n* = 1)	—
Group II	27.90 ± 3.20 (*n* = 4)	26.62 ± 2.84 (*n* = 4)	25.45 ± 1.87 (*n* = 4)	28.23 ± 1.22 (*n* = 4)	31.25 ± 0.92 (*n* = 4)	36.40 ± 0.00 (*n* = 1)
Group III	23.35 ± 3.80 (*n* = 4)	26.38 ± 3.32 (*n* = 4)	29.42 ± 4.27 (*n* = 4)	26.07 ± 2.48 (*n* = 3)	26.73 ± 2.71 (*n* = 3)	22.00 ± 0.00 (*n* = 1)
Group IV	29.98 ± 1.03 (*n* = 4)	30.63 ± 1.74 (*n* = 4)	28.05 ± 5.24 (*n* = 4)	20.00 ± 3.8 (*n* = 2)	19.80 ± 0.00 (*n* = 1)	—
Group V	32.28 ± 1.71 (*n* = 4)	33.70 ± 1.85 (*n* = 4)	35.55 ± 1.94 (*n* = 4)	38.20 ± 1.40 (*n* = 2)	—	—

TEC (×10^12^/L)						
Group I	4.18 ± 0.22 (*n* = 4)	4.65 ± 0.17 (*n* = 4)	4.99 ± 0.07 (*n* = 4)	5.20 ± 0.17 (*n* = 4)	4.92 ± 0.00 (*n* = 1)	—
Group II	3.95 ± 0.56 (*n* = 4)	3.73 ± 0.61 (*n* = 4)	3.54 ± 0.50 (*n* = 4)	3.94 ± 0.37 (*n* = 4)	4.44 ± 0.26 (*n* = 4)	5.12 ± 0.00 (*n* = 1)
Group III	3.48 ± 0.56 (*n* = 4)	3.85 ± 0.38 (*n* = 4)	4.08 ± 0.26 (*n* = 4)	3.92 ± 0.47 (*n* = 3)	4.27 ± 0.32 (*n* = 3)	3.95 ± 0.00 (*n* = 1)
Group IV	4.37 ± 0.23 (*n* = 4)	4.48 ± 0.31 (*n* = 4)	4.56 ± 0.82 (*n* = 4)	3.46 ± 1.44 (*n* = 2)	2.32 ± 0.00 (*n* = 1)	—
Group V	4.89 ± 0.34 (*n* = 4)	5.00 ± 0.31 (*n* = 4)	5.11 ± 0.35 (*n* = 4)	4.97 ± 0.99 (*n* = 2)	—	—

**Table 6:** Total leukocyte counts (×10^9^/L) in different groups at various observation intervals (mean ± S.E.).

Groups	Days					
	0	3	6	9	12	15
Group I	25.10 ± 4.33	23.30 ± 2.05	17.87 ± 3.79	17.33 ± 2.54	13.2 ± 0.00 (*n* = 1)	—
Group II	34.18 ± 4.39	25.18 ± 4.61	15.22** ± 2.50	12.95** ± 3.10	11.15** ± 2.10	6.9 ± 0.00 (*n* = 1)
Group III	31.95 ± 4.68	21.83* ± 2.59	11.63** ± 2.61	11.47** ± 3.34 (*n* = 3)	9.07** ± 1.37 (*n* = 3)	9.1 ± 0.00 (*n* = 1)
Group IV	28.85 ± 1.95	18.23* ± 5.00	11.23** ± 1.38	10.65 ± 1.25 (*n* = 2)	11.80 ± 0.00 (*n* = 1)	—
Group V	28.03 ± 1.98	21.73* ± 1.40	14.48** ± 1.84	7.85 ± 1.05 (*n* = 2)	—	—

*P < 0.05, **P < 0.01.

**Table 7:** Differential leukocyte count (%) in dogs of different groups at various observation intervals (mean ± S.E.).

Groups	Days					
	0	3	6	9	12	15
Granulocytes (%)						
Group I	88.73 ± 1.01	83.65 ± 0.64	74.92 ± 3.69	71.23 ± 5.67	86.60 ± 0.00 (*n* = 1)	—
Group II	91.10 ± 1.91	86.97 ± 1.31	84.50 ± 1.87	80.5 ± 1.59	78.42 ± 2.86	81.9 ± 0.00 (*n* = 1)
Group III	88.57 ± 1.55	84.43 ± 2.86	79.90* ± 2.14	75.5** ± 1.15 (*n* = 3)	71.33** ± 1.18 (*n* = 3)	67.00 ± 0.00 (*n* = 1)
Group IV	85.48 ± 3.68	87.05 ± 3.00	78.28 ± 3.42	65.00 ± 7.10 (*n* = 2)	50.80 ± 0.00 (*n* = 1)	—
Group V	89.18 ± 0.79	84.40 ± 3.36	80.33 ± 2.28	76.4 ± 9.60 (*n* = 2)	—	—

Lymphocytes (%)						
Group I	8.73 ± 1.06	13.57 ± 0.81	21.45 ± 3.16	18.93 ± 0.07	11.20 ± 0.00 (*n* = 1)	—
Group II	6.66 ± 1.44	9.73 ± 1.57	11.42 ± 1.02	15.00 ± 1.54	14.35 ± 1.63	12.60 ± 0.00 (*n* = 1)
Group III	8.40 ± 1.21	12.43 ± 2.82	16.45 ± 2.08	20.20 ± 1.21 (*n* = 3)	20.70 ± 3.61 (*n* = 3)	28.60 ± 0.00 (*n* = 1)
Group IV	10.58 ± 2.15	10.03 ± 2.75	17.18 ± 2.70	28.70 ± 7.80 (*n* = 2)	36.60 ± 0.00 (*n* = 1)	—
Group V	8.35 ± 0.55	12.83** ± 1.41	16.35** ± 0.64	20.85 ± 4.15 (*n* = 2)	—	—

Monocytes (%)						
Group I	2.55 ± 0.26	2.80 ± 0.29	3.93 ± 0.97	4.25 ± 1.25	2.20 ± 0.00 (*n* = 1)	—
Group II	2.25 ± 0.48	3.10 ± 0.60	3.58 ± 0.75	4.83 ± 1.01	5.75 ± 1.13	5.50 ± 0.00 (*n* = 1)
Group III	3.02 ± 0.38	3.15 ± 0.30	3.65 ± 0.25	4.30 ± 0.15 (*n* = 3)	4.60 ± 0.71 (*n* = 3)	4.40 ± 0.00 (*n* = 1)
Group IV	3.20 ± 0.92	2.93 ± 0.38	4.30 ± 0.63	5.75 ± 0.15 (*n* = 2)	5.60 ± 0.00 (*n* = 1)	—
Group V	2.40 ± 0.36	2.78 ± 0.26	4.63 ± 0.99	5.70 ± 2.40 (*n* = 2)	—	—

*P < 0.05, **P < 0.01.

**Table 8:** AST and ALT in different groups at various observation intervals (mean ± S.E.).

Groups	Days				
	0	7	14	21	28
AST (U/L)					
Group I	28.75 ± 3.09	31.00 ± 4.85	36.00 ± 10.42	27.25 ± 4.31	—
Group II	25.75 ± 6.42	39.75 ± 5.36	38.25 ± 8.35	31.75 ± 8.67	42.00 ± 0.00
Group III	26.25 ± 4.39	34.5 ± 4.27	30.75 ± 4.3	29 ± 4.38	—
Group IV	33.5 ± 9.33	38.75 ± 5.98	42.25 ± 8.02	32.33 ± 5.67	—
Group V	28 ± 7.93	31.5 ± 5.12	35.00 ± 9.58	33.67 ± 1.67	—

ALT (U/L)					
Group I	31.75 ± 6.96	58 ± 18.37	45.25 ± 9.91	33.5 ± 3.96	—
Group II	36.25 ± 7.31	45.75 ± 5.72	35.5 ± 7.04	45.76 ± 3.88	30 ± 0.00
Group III	41 ± 7.69	44.5 ± 8.03	45.5 ± 3.59	49.5 ± 2.99	—
Group IV	29 ± 4.69	30.75 ± 5.37	45.75 ± 9.92	43 ± 2.65	—
Group V	23.2 ± 2.50	27.27 ± 3.66	41.25 ± 8.71	27.25 ± 3.33	—
